# Quantitative Cerebrovascular Reactivity in Normal Aging: Comparison Between Phase-Contrast and Arterial Spin Labeling MRI

**DOI:** 10.3389/fneur.2020.00758

**Published:** 2020-07-31

**Authors:** Kamil Taneja, Peiying Liu, Cuimei Xu, Monroe Turner, Yuguang Zhao, Dema Abdelkarim, Binu P. Thomas, Bart Rypma, Hanzhang Lu

**Affiliations:** ^1^The Russel H. Morgan Department of Radiology, Johns Hopkins University School of Medicine, Baltimore, MD, United States; ^2^School of Behavioral and Brain Sciences, University of Texas at Dallas, Richardson, TX, United States; ^3^Advanced Imaging Research Center, University of Texas Southwestern Medical Center, Dallas, TX, United States; ^4^Department of Psychiatry, University of Texas Southwestern Medical Center, Dallas, TX, United States; ^5^F.M. Kirby Research Center for Functional Brain Imaging, Kennedy Krieger Institute, Baltimore, MD, United States; ^6^Department of Biomedical Engineering, Johns Hopkins University School of Medicine, Baltimore, MD, United States

**Keywords:** phase-contrast, arterial spin labeling, MRI, cerebrovascular reactivity, aging

## Abstract

**Purpose:** Cerebrovascular reactivity (CVR) is an index of the dilatory function of cerebral blood vessels and has shown great promise in the diagnosis of risk factors in cerebrovascular disease. Aging is one such risk factor; thus, it is important to characterize age-related differences in CVR. CVR can be measured by BOLD MRI but few studies have measured quantitative cerebral blood flow (CBF)-based CVR in the context of aging. This study aims to determine the age effect on CVR using two quantitative CBF techniques, phase-contrast (PC), and arterial spin labeling (ASL) MRI.

**Methods:** In 49 participants (32 younger and 17 older), CVR was measured with PC, ASL, and BOLD MRI. These CVR methods were compared across young and older groups to determine their dependence on age. PC and ASL CVR were also studied for inter-correlation and mean differences. Gray and white matter CVR values were also studied.

**Results:** PC CVR was higher in younger participants than older participants (by 17%, *p* = 0.046). However, there were no age differences in ASL or BOLD CVR. ASL CVR was significantly correlated with PC CVR (*p* = 0.042) and BOLD CVR (*p* = 0.016), but its values were underestimated compared to PC CVR (*p* = 0.045). ASL CVR map revealed no difference between gray matter and white matter tissue types, whereas gray matter was significantly higher than white matter in the BOLD CVR map.

**Conclusion:** This study compared two quantitative CVR techniques in the context of brain aging and revealed that PC CVR is a more sensitive method for detection of age differences, despite the absence of spatial information. The ASL method showed a significant correlation with PC and BOLD, but it tends to underestimate CVR due to confounding factors associated with this technique. Importantly, our data suggest that there is not a difference in CBF-based CVR between the gray and white matter, in contrast to previous observation using BOLD MRI.

## Introduction

Cerebrovascular reactivity (CVR) is an index of the dilatory function of cerebral blood vessels. This index has potential utility in arterial stenosis (1–3), stroke (4–6), neurodegenerative diseases (7–9), and normal aging (10–12). CVR can be measured by applying a vasodilatory challenge, such as CO_2_ (13), while recording hemodynamic parameters of the brain, and the change in the hemodynamic parameter per unit dilatory stimulus is quantified as CVR.

The vast majority of prior CVR studies have been based on the Blood-Oxygenation-Level-Dependent (BOLD) MRI signal (2, 10, 12–14), due to its straightforward pulse sequence, high sensitivity, high spatial, and temporal resolution (13). However, BOLD signal reflects a complex interplay between many physiological parameters such as cerebral blood flow (CBF), venous cerebral blood volume (vCBV), cerebral metabolic rate of oxygen (CMRO_2_), and hematocrit (Hct) (14), thus this signal is often considered as a semi-quantitative measure of brain hemodynamics and its interpretation is sometimes difficult (15–17).

CBF-based CVR is generally considered a more quantitative measure. This technique has a direct physiological meaning, thus is not dependent on magnetic field strength. Furthermore, such a measure can be compared across imaging modalities such as MRI (18), Positron Emission Tomography (PET) (19), Single Photon Emission Computed Tomography (SPECT) (20), ultrasound (21), and optical imaging (22). Two commonly used MRI approaches to measure CBF are arterial spin labeling (ASL) and phase-contrast (PC) MRI. ASL measures voxel-wise CBF by using labeled water protons as an endogenous tracer (18, 23, 24) and subtraction of label and control images yields a perfusion-weighted image, which can be used for quantification of CBF maps (25, 26). Two major limitations of ASL are that it suffers from low signal-to-noise ratio (SNR) and that there exist several confounding factors in its quantification such as bolus arrival time and labeling efficiency (27). These factors are known to vary with CO2 levels (28, 29).

PC MRI is a technique to measure blood flow via imaging of flow velocity in blood vessels (28, 30). This method can be used to measure global CBF through arterial vessels (e.g., internal carotid arteries) or venous vessels (e.g., superior sagittal sinus, SSS). PC MRI does not suffer from variables such as bolus arrival time or labeling efficiency, although it does not provide regional CBF information. CBF measurements from PC and ASL MRI under basal states have been compared previously (13, 27, 28).

The purpose of this study was to compare PC and ASL-based CVR in the context of normal aging. For completeness, BOLD-based CVR data were also collected. CVR was measured in younger (*N* = 32) and older (*N* = 17) participants with all three techniques to determine their sensitivity to age-related cerebrovascular decline. The correlation and magnitude between these CVR techniques were also studied. Basal CBF differences between the younger and older groups were examined using the normocapnic PC data.

## Materials and Methods

### Study Participants

Forty-nine participants (27 females, 22 males) were recruited at the University of Texas at Dallas (UTD) and Johns Hopkins University (JHU). The demographic and cognitive information of participants from both institutions is summarized in Table 1. The study was approved by the Institutional Review Boards of both institutions. Written informed consent in accordance with the Declaration of Helsinki was obtained before participants were enrolled in the study.

**Table 1:** Demographic and cognitive information for study participants.

	**Young**	**Old**	***p***	**t_44_**
**Demographic Information**				
***N***	32	17		
UTD	16	10		
JHU	16	7		
**Age, years (SE)**				
UTD	23.1 (0.6)	59.6 (1.6)		
JHU	20.9 (0.6)	66.0 (3.0)		
**Gender (M, F)**				
UTD	(5, 11)	(3, 7)		
JHU	(11, 5)	(3, 4)		
**Cognitive Measures**, ***z*****-Score (SE)**				
Multiple domains	−0.007 (0.206)	0.038 (0.129)	1	0
Motor speed	0.312 (0.139)	−0.404 (0.198)	0.01	−2.69
Attention	0.118 (0.165)	−0.117 (0.194)	1	0
Short term memory	0.356 (0.162)	−0.543 (0.163)	0.141	−1.50
Working memory	0.233 (0.186)	−0.372 (0.149)	0.692	−0.40
Fluid intelligence	0.328 (0.132)	−0.519 (0.208)	0.019	−2.44
Crystalline intelligence	0.182 (0.117)	−0.336 (0.241)	0.37	−0.91
Processing speed	0.475 (0.112)	−0.767 (0.112)	<0.001	−5.86
Global cognition	0.328 (0.085)	−0.510 (0.111)	<0.001	−4.66

*F, female; JHU, Johns Hopkins University; M, male; N, number of participants; SE, standard error; UTD, University of Texas at Dallas*.

### Experimental Procedures

**Figure 1:**
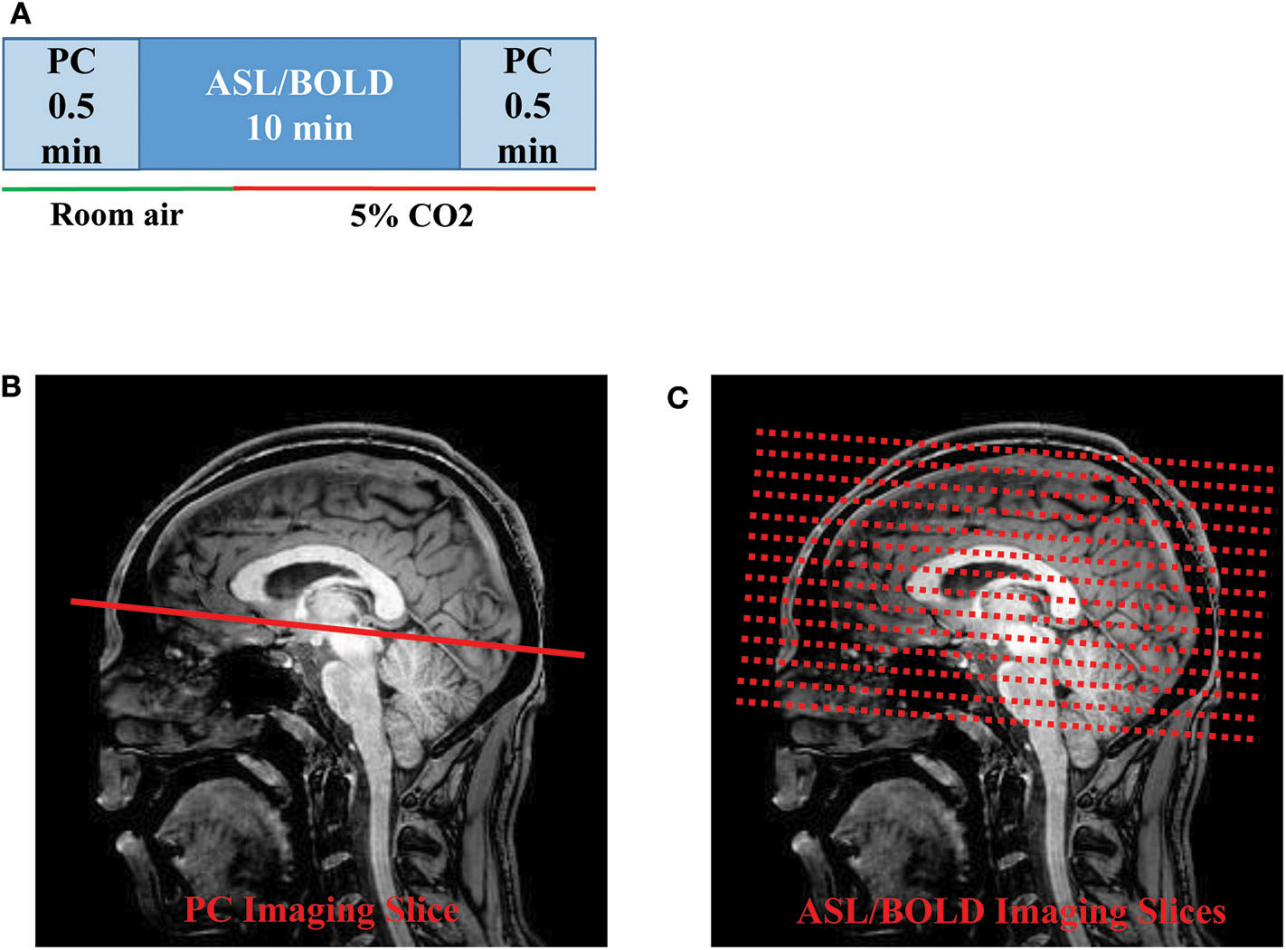
Illustration of the MRI scan procedures: **(A)** Timing of the PC and ASL/BOLD MRI scans during normocapnic and hypercapnic states. The timing of the gas switch is shown by the colored bars. The gas switch occurred 4 min into the ASL/BOLD scan. **(B)** Slice positioning of PC and **(C)** ASL/BOLD MRI. ASL, arterial spin labeling; BOLD, blood-oxygenation-level-dependent; PC, phase-contrast.

The MRI scans were performed on two 3T systems (Philips Healthcare, Best, The Netherlands). The two systems have the identical model number (Achieva with QUASAR gradient) and hardware configurations. A body coil was used for transmission and a 32-channel head coil was used for receiving. Before the subject entered the scanner, a nose clip and a mouth piece was attached on the subject to allow for control of inspired air, as detailed previously (31). Once the subject was inside the scanner, we performed MRI scans illustrated in Figure 1A. The scan session began with room air breathing. A PC-MRI was performed first followed by a dual-echo ASL/BOLD sequence for 10 min. At 4 min into the ASL/BOLD sequence (green line in Figure 1A), the inspired gas was switched to CO_2_-enriched gas mixture (5% CO_2_, 21% O_2_, and 74% N_2_) and the scan continued for another 6 min. The CO_2_ gas mixture was stored in a Douglas bag and delivered through a two-way non-rebreathing valve (Hans Rudolph, 2600 series, Shawnee, KS). A research assistant was in the MRI room to change the valve from room-air to 5% CO_2_ gas at the appropriate time. After the dual-echo ASL/BOLD sequence, a PC-MRI was performed in the hypercapnic state. End-tidal CO_2_ (EtCO_2_) was continuously measured through the duration of the experiment using a capnograph device (NM3 Respiratory Profile Monitor, Model 7900, Philips Healthcare, Wallingford, CT).

PC-MRI used the following parameters: field-of-view (FOV) = 230 × 230 mm^2^, matrix size = 320 × 192, slice thickness = 5 mm, repetition time (TR)/echo time (TE)/flip angle (FA) = 20 ms/7 ms/15°, velocity encoding (V_enc_) = 80 cm/s, scan duration= 30 s. The 2D PC imaging slice was positioned at 20 mm above sinus confluence with the imaging slice parallel to AC-PC line to measure flow through the SSS (Figure 1B). The dual-echo ASL sequence used the following parameters: pseudo-continuous ASL (pCASL) labeling, FOV = 220 × 220 × 126 mm^3^, matrix size = 64 × 64 × 22, slice thickness = 6 mm, TR/TE1/TE2/FA = 4,006 ms/13 ms/30 ms/90°, scan duration = 10 min (Figure 1C). The sequence was used to obtain simultaneous ASL (the TE1 data) and BOLD (the TE2 data) signals. Note, however, that these BOLD data are slightly different from the traditional BOLD-CVR data which are typically acquired in several CO_2_ breathing cycles with a shorter breathing period (13).

A T1-weighted magnetization-prepared rapid acquisition with gradient echo (MPRAGE) scan was performed for tissue segmentation. The MPRAGE sequence had the following parameters: TR of 8.1 ms, TE of 3.7 ms, a flip angle of 12°, a shot interval of 2,100 ms, an inversion time (TI) of 1,100 ms, with a voxel size of 1 × 1 × 1 mm^3^, 160 slices with a sagittal slice orientation.

### Data Processing

Data analysis was performed using Statistical Parameter Mapping (SPM) (University College London, UK) and in-house MATLAB scripts (MathWorks, Natick, MA).

**Figure 2:**
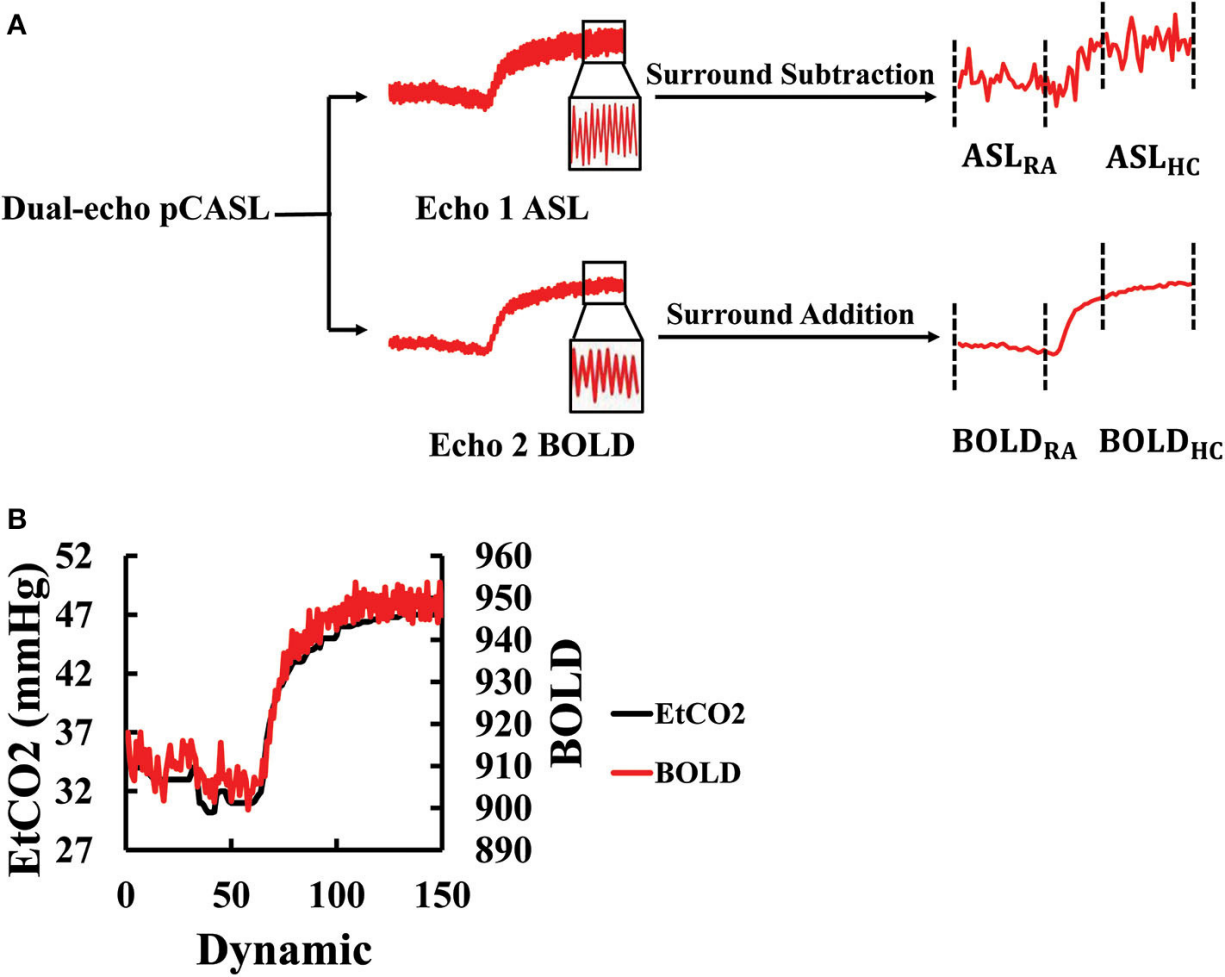
Processing of the dual-echo ASL/BOLD MRI data: **(A)** Flowchart of the ASL/BOLD data processing. The dual-echo data were first split into two echoes, the first corresponding to ASL and the second BOLD. The time courses of these raw data reveal high-frequency signal fluctuations in accordance with the control-label alternations. Subtraction and addition of the time courses result in the final ASL and BOLD time courses, respectively. The corresponding time periods were averaged to obtain room-air (RA) and hypercapnic (HC) signal values. **(B)** Illustration of the maximal alignment between EtCO_2_ and WB BOLD signal from a representative subject. The EtCO_2_ time course was shifted to find the best temporal match with the BOLD. ASL, arterial spin labeling; BOLD, blood-oxygen-level-dependent; EtCO_2_, end-tidal CO_2_; HC, hypercapnia; pCASL, pseudo-continuous arterial spin labeling; RA, room air; WB, whole-brain.

The dual-echo ASL scan yielded both ASL and BOLD data. The ASL (first echo) and BOLD (second echo) images were first preprocessed using a standard pipeline: motion correction by realignment (to the first dynamic) and spatial smoothing with a Gaussian filter with a full-width-at-half-maximum of 4 mm. A whole-brain (WB) mask was obtained by segmentation of the raw image and used to obtain a WB raw ASL and BOLD time course. The ASL label and control values were subtracted using a surround subtraction algorithm (32, 33) to obtain CBF-weighted time course (Figure 2A). The BOLD label and control values were averaged to cancel out the labeling effect (34), yielding a final BOLD time course (Figure 2A). Note that the dual-echo ASL scan lasted for 10 min with the switch from room air to CO_2_ gas taking place at 4 min into the scan. We averaged the data in the first 4 min as the room air values (ASL_RA_ and BOLD_RA_) and the last 4 min as the hypercapnic values (ASL_HC_ and BOLD_HC_) (Figure 2A). Data during the middle 2 min were not used for CVR calculation as physiology was not in a steady-state.

ASL and BOLD based CVR were then calculated with:

(1) $$\begin{array}{l} \text{ASL CVR}=\;\frac{100*\frac{{\text{ASL}}_{\text{HC}}-{\text{ASL}}_{\text{RA}}}{{\text{ASL}}_{\text{RA}}}}{{\text{EtCO}2}_{\text{ ASL},\text{HC}}-{\text{EtCO}2}_{\text{ASL},\text{ RA}}} \end{array}$$

(2) $$\begin{array}{l} \text{BOLD CVR}=\;\frac{100*\frac{{\text{BOLD}}_{\text{HC}}-{\text{BOLD}}_{\text{RA}}}{{\text{BOLD}}_{\text{RA}}}}{{\text{EtCO}2}_{\text{ASL},\text{ HC}}-{\text{EtCO}2}_{\text{ASL},\text{ RA}}} \end{array}$$

where EtCO_2HC_ and EtCO_2RA_ represent end-tidal CO_2_ values during the hypercapnia and room air MRI acquisition, respectively.

The EtCO_2_ values were extracted from the CO_2_ recording. To obtain EtCO_2HC_ and EtCO_2RA_ that are time-matched to the MRI data acquisitions, the EtCO_2_ time course was temporally aligned to the BOLD signal time course by computing cross-correlation (CC) between them at various time-shifts and finding the shift yielding the highest CC (Figure 2B). This process allows the correction of the time it takes for the CO_2_ to travel from the lungs to the brain (13). Once temporally aligned, the EtCO_2_ data points during the corresponding MRI scan were averaged to yield EtCO_2_ values room air and hypercapnia, which was used for the calculation of CVR in Equations (1) and (2).

In addition to WB CVR values, which include both gray matter (GM) and white matter (WM), we also separately examined GM and WM CVR. To create GM and WM masks, the T1-weighted MPRAGE image was skull-stripped and segmented using SPM12.

**Figure 3:**
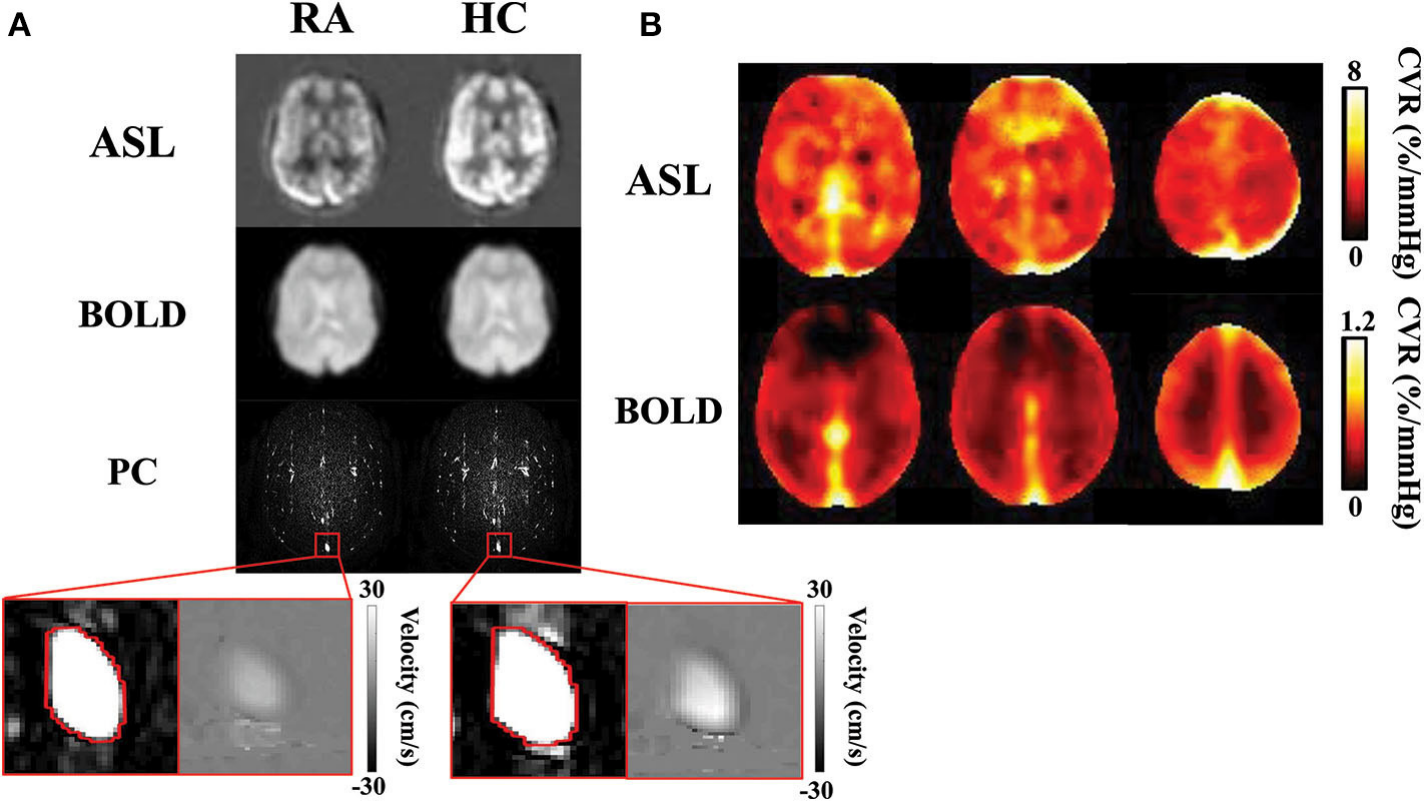
CVR results obtained with ASL, BOLD, and PC MRI data: **(A)** Normocapnic and hypercapnic ASL, BOLD, and PC images from a representative subject. For PC MRI, the complex difference images are shown, with a zoom-in version showing the complex difference and velocity map in the superior sagittal sinus. **(B)** Group-average ASL and BOLD CVR maps. Note that the ASL and BOLD CVR values have different ranges. ASL, arterial spin labeling; BOLD, blood-oxygen-level-dependent; CVR, cerebrovascular reactivity; HC, hypercapnia; PC, phase-contrast; RA, room air.

To calculate PC CVR, the flux at room air (*Flux*_*RA*_) and hypercapnia (*Flux*_*HC*_) were measured. Region-of-interests (ROIs) encompassing the SSS were manually drawn on complex difference images for both the room air and hypercapnia PC scans (Figure 3A). These ROIs were then overlaid onto the respective velocity maps, and flux was calculated by the multiplying the ROI area by the mean velocity in the ROI. PC CVR could then be calculated as:

(3) $$\begin{array}{l} \text{PC CVR}=\;\frac{100*\frac{{\text{Flux}}_{\text{HC}}-{\text{Flux}}_{\text{RA}}}{{\text{Flux}}_{\text{RA}}}}{{\text{EtCO}2}_{\text{PC},\text{ HC}}-{\text{EtCO}2}_{\text{PC},\text{ RA}}} \end{array}$$

where EtCO_2PC, HC_ and EtCO_2PC, RA_ represent averaged end-tidal CO_2_ values during hypercapnic and room air PC scan, respectively.

The PC MRI scan during room air breathing was also used to calculate basal CBF (ml/100 g/min):

(4) $$\begin{array}{l} \text{Basal CBF}=\frac{{\text{Flux}}_{\text{RA}}}{\text{Brain Volume }*1.06\;*100\;}*\frac{1}{0.46} \end{array}$$

where 0.46 represents the ratio between flow in the SSS and that in the whole-brain (35), brain volume was estimated by segmentation of the T1-MPRAGE image using MRIcloud (36). 1.06 is the mass density of brain tissue (37), and the number “100” allows the expression of CBF in “per 100 g tissue.”

### Statistical Analysis

All CVR values are reported as mean ± standard error. CVR was compared across young and older groups with a linear regression analysis, after adjusting for sex and site. For comparison of CVR across MRI techniques, a Pearson correlation was computed. For within-subject comparison of tissue type effect and imaging method effect, a paired *t*-test was used. A *p*-value of 0.05 or less was considered significant.

## Results

Figure 3A illustrates images of PC, ASL, and BOLD MR images during room air and hypercapnia breathing from a representative subject. Group-averaged ASL and BOLD-based CVR maps are shown in Figure 3B. Note that the ASL CVR map is noisier compared to the BOLD CVR map, consistent with a lower SNR of the ASL data. Importantly, the ASL CVR map shows an absence of GM-WM contrast. This is confirmed by region-of-interest analysis which showed that GM and WM CVR were not different in the ASL data (GM ASL CVR = 5.6 ± 0.3% /mmHg, WM ASL CVR = 5.1 ± 0.4 %/mmHg, *p* > 0.05, *t*_48_ = 1.9). This observation suggests that, even though GM contains more blood vessels (thereby higher basal perfusion) than WM, their vascular reactivities in terms of fractional changes in CBF are comparable. On the other hand, the BOLD CVR map shows a higher CVR in the GM than WM, due to the confounding factors (e.g., vCBV) in the BOLD signal (38). Accordingly, GM BOLD CVR was found to be significantly higher than WM BOLD CVR (GM = 0.33 ± 0.01 %/mmHg, WM = 0.19 ± 0.01 %/mmHg, *p* < 0.001, *t*_48_ = 15.1). Note that PC CVR is a global measure and does not produce a map.

Table 2 summarizes the age effect on the three CVR modalities. Only PC CVR demonstrates an age effect, where older participants (5.46 ± 0.32 %/mmHg) have a significantly lower PC CVR than younger participants (6.59 ± 0.41 %/mmHg, *p* = 0.046, *t*_45_ = −2.06). On the other hand, WB ASL CVR (young = 5.53 ± 0.37 %/mmHg, old = 5.03 ± 0.55 %/mmHg, *p* = 0.31, *t*_45_ = −1.03), GM ASL CVR (young = 5.74 ± 0.35 %/mmHg, old = 5.31± 0.6 %/mmHg, *p* = 0.35, *t*_45_ = −0.95), and WM ASL CVR (young = 5.27 ± 0.53 %/mmHg, old = 4.86 ± 0.58 %/mmHg, *p* = 0.52, *t*_45_ = −0.65) did not display a significant age effect. Similarly, WB BOLD CVR (young = 0.27 ± 0.01 %/mmHg, old= 0.28 ± 0.01 %/mmHg, *p* = 0.72, *t*_45_ = 0.37), GM BOLD CVR (young = 0.33 ± 0.01 %/mmHg, old = 0.33 ± 0.01 %/mmHg, *p* = 0.95, *t*_45_ = 0.07 ), and WM BOLD CVR (young = 0.19 ± 0.01 %/mmHg, old = 0.21 ± 0.02 %/mmHg, *p* = 0.29, *t*_45_ = 1.07) did not demonstrate a significant age effect.

**Table 2:** CVR age differences.

	**Young (%/mmHg)**	**Old (%/mmHg)**	***p***	**t_44_**
**WB CVR, (SE)**				
PC	6.59 (0.41)	5.46 (0.32)	0.046	−2.06
ASL	5.53 (0.37)	5.03 (0.55)	0.31	−1.03
BOLD	0.27 (0.01)	0.28 (0.01)	0.72	0.37
**GM CVR, (SE)**				
ASL	5.74 (0.35)	5.31 (0.60)	0.35	−0.95
BOLD	0.33 (0.01)	0.33 (0.01)	0.95	0.07
**WM CVR, (SE)**				
ASL	5.27 (0.53)	4.86 (0.58)	0.52	−0.65
BOLD	0.19 (0.01)	0.21 (0.02)	0.29	1.07

*ASL, arterial spin labeling; BOLD, blood-oxygen-level-dependent; CVR, cerebrovascular reactivity; GM, gray matter; PC, phase-contrast; SE, standard error; WB, whole-brain; WM, white matter*.

Since both PC and ASL MRI measures CBF-based CVR, we compared whole-brain values between these two imaging methods. It was found that WB ASL CVR (5.37 ± 0.32 %/mmHg) was significantly lower than PC CVR (6.15 ± 0.30 %/mmHg, *p* = 0.045, *t*_48_ = 2.06), consistent with previous suggestions that confounding factors in ASL quantification (e.g., reduced labeling efficiency during hypercapnia) may result in a bias in CVR values when measured with ASL MRI.

**Figure 4:**
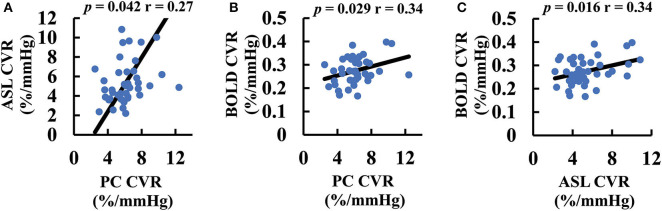
Scatter plots between CVR values measured with different MRI techniques: **(A)** ASL and PC CVR. **(B)** BOLD and PC CVR. **(C)** BOLD and ASL CVR. The black lines are the fitting curves. The *p*-values associated with their correlations are also shown. ASL, arterial spin labeling; BOLD, blood-oxygen-level-dependent; CVR, cerebrovascular reactivity; GM, gray matter; PC, phase-contrast; WB, whole-brain; WM, white-matter.

We further studied the correlation between different CVR methods across participants. Figure 4 shows correlations between the different CVR measures. Despite different pulse sequences and different underlying physiological mechanisms, all three CVR methods showed a significant correlation (*p* < 0.05).

**Figure 5:**
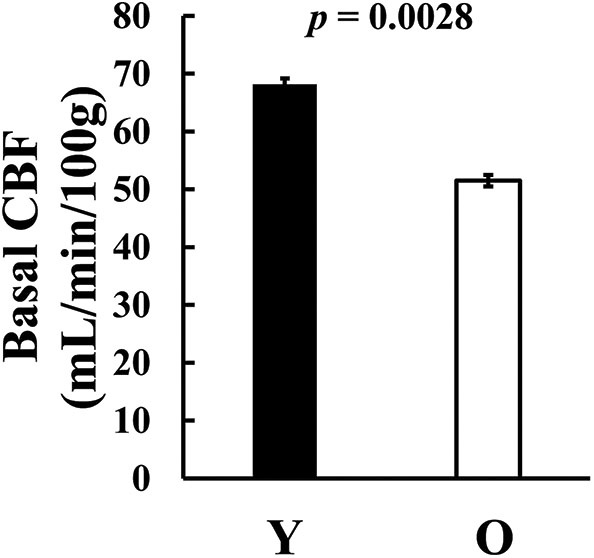
Basal CBF difference between age groups. CBF, cerebral blood flow; O, old; Y, young.

We also examined basal CBF, (i.e., CBF without hypercapnia challenge). We found that older subjects revealed a significantly lower CBF compared to the younger group (young = 68.2 ± 3.9 ml/100 g/min, old = 51.5 ± 3.5 ml/100 g/min, *p* = 0.0028, *t*_44_ = 3.17, Figure 5), consistent with previous reports (10, 38–40).

## Discussion

CVR is known to be a sensitive marker in large vessel diseases, such as Moyamoya disease and stroke (1, 6, 41, 42), and small vessel diseases, such as vascular cognitive impairment (43, 44) and dementia (7, 45) and diabetes (46, 47). Because aging is a risk factor for many of these diseases, it is important to characterize age-related changes in CVR. Several studies have investigated this topic using BOLD signal as the readout for hemodynamic responses (10–12, 48). However, BOLD signal reflects a complex interplay between vCBV, CBF, and CMRO_2_, thus its quantitative interpretation is not trivial, both in terms of brain physiology (13, 14) and age-related physiological changes [e.g., (34)]. Furthermore, there is a non-linear relationship between blood CO_2_ content and BOLD signal change (49, 50). Therefore, a more direct and less complex measurement of brain hemodynamics is desirable to deliver more quantitative measurements of CVR. PC MRI and ASL MRI represent two of the most commonly used CBF techniques, both of which require no contrast agent and are suitable for dynamic measurements. ASL measures microscopic perfusion in a region-specific manner. However, ASL has a few disadvantages including lower SNR and its susceptibility to several confounding factors such as bolus arrival time (51) and labeling efficiency (28), which are known to change during hypercapnia. On the other hand, PC MRI measures macroscopic flow velocity in large vessels (e.g., superior sagittal sinus, internal carotid artery) and the pulse sequence is relatively straightforward with few confounding factors. However, this technique provides only a global measure and does not provide spatial information. Data from the present study suggested that, although lacking regional information, PC CVR was able to detect age-related decreases in CVR, which may be attributed to changes such as higher vessel stiffness, increased basement membrane thickness, reduced smooth muscle volume, and impaired astrocyte, or endothelial function (52, 53). It is worth noting that ASL CVR significantly underestimated CVR, presumably due to reduced labeling efficiency during the hypercapnic state (28, 54), and was unable to identify a significant age reduction. Therefore, for global assessment, PC CVR provides a sensitive and reliable measurement of cerebrovascular function. It should also be noted that PC MRI scan only takes 0.5 min for each state, thus is a rapid pulse sequence compared to ASL or BOLD MRI.

An interesting observation of the present study is that we showed, for the first time, a voxel-wise ASL CVR map, which revealed that GM and WM evince similar CVR. It is known that WM has substantially lower vessel density compared to the GM (55), often yielding substantially lower WM hemodynamic parameters than those in GM. This includes vCBV, CBF, and CMRO_2_ (39, 56–59). Previous studies of BOLD CVR have also shown a clear gray-white contrast, with GM having a higher CVR than WM (2, 10, 12–14). Our results suggest the hypothesis that this gray-white matter difference might be artifactual, due to the complexity of the physiology from which the BOLD signal arises. The results of the present study suggest that there is not a clear gray-white matter difference in ASL-CVR. Indeed, ROI results confirmed this finding, revealing equivalent CVR values in these two tissue types. This is physiologically plausible in that, although CO_2_ induced CBF change is lower in WM, its basal CBF is also lower. Thus, CVR based on fractional changes in CBF is actually similar between GM and WM.

Our conclusions are tempered by some limitations in this study. First, a step-breathing paradigm was used in the BOLD CVR scan instead of the more common block-cycle breathing paradigm. Step-breathing was used because the BOLD CVR data were collected simultaneously with the ASL CVR data. A potential problem with the step breathing paradigm is that it is not possible to correct any BOLD signal drifts due to gradient heating or electronic instabilities (60, 61). Thus, the lack of age effect might be partly attributed to the non-traditional BOLD paradigm used. Another possible reason for the lack of age difference in BOLD CVR is that, due to the nature of the pCASL sequence (e.g., long labeling duration, long post-labeling delay, and the need for one control and one labeled image), the BOLD data in the present study had a temporal resolution of 8 s, which is considerably longer than typical BOLD acquisition of <2 s. This may have reduced the sensitivity of the BOLD data and resulted in an absence of age effect. Another limitation is that the pCASL sequence used a multi-slice 2D EPI, rather than 3D GRASE or stack-of-spiral, acquisition. This is to allow T2^*^ contrast to be preserved in the signal which is important for the BOLD signal. However, 3D acquisition would have yielded a higher SNR. A final limitation is that the data presented in this work were collected at two different sites, due to our study design. However, we note that the MRI scanners used at the two sites were identical in terms of hardware and software configurations and that the gas-inhalation apparatus was also identical (31).

## Conclusion

This study compared two quantitative CVR techniques in the context of brain aging and revealed that PC CVR is a more sensitive method to detect age differences, despite a lack of spatial information. The ASL method showed a significant correlation with PC and BOLD, but it tends to underestimate CVR due to confounding factors associated with this technique. Importantly, our data suggest that there is not a difference in CBF-based CVR between GM and WM, in contrast to previous observation using BOLD MRI.

## Data Availability Statement

The raw data supporting the conclusions of this article will be made available by the authors, without undue reservation.

## Ethics Statement

The studies involving human participants were reviewed and approved by Johns Hopkins University IRB; UT Southwestern IRB. The patients/participants provided their written informed consent to participate in this study.

## Author Contributions

BT and BR were involved with study design. CX, DA, and YZ were involved with data collection. HL was involved with study design and manuscript writing. KT was involved with data collection, data analysis, and manuscript writing. PL and MT were involved with study design and data collection. All authors commented on the manuscript, read, and approved the final version.

## Conflict of Interest

The authors declare that the research was conducted in the absence of any commercial or financial relationships that could be construed as a potential conflict of interest.

## Abbreviations

ASL: arterial spin labeling
*ASL*_*RA*_: room air arterial spin labeling
*ASL*_*HC*_: room air arterial spin labeling
BOLD: blood-oxygen-level-dependent
*BOLD*_*RA*_: room air blood-oxygen-level-dependent
*BOLD*_*HC*_: hypercapnia blood-oxygen-level-dependent
CBF: cerebral blood flow
CC: cross correlation
CVR: cerebrovascular reactivity
EtCO_2_: end-tidal CO_2_
*EtCO*2_*ASL, RA*_: end-tidal CO_2_ during arterial spin labeling at room air
*EtCO*2_*ASL, HC*_: end-tidal CO_2_ during arterial spin labeling at hypercapnia
*EtCO*2_*PC, RA*_: end-tidal CO_2_ during phase-contrast at room air
*EtCO*2_*PC, HC*_: end-tidal CO_2_ during phase-contrast at hypercapnia
FA: flip angle
*Flux*_*RA*_: flux at room air
*Flux*_*HC*_: flux at hypercapnia
FOV: field-of-view
GM: gray matter
HC: hypercapnia
Hct: hematocrit
JHU: Johns Hopkins University
MPRAGE: magnetization-prepared rapid acquisition with gradient echo
PC: phase-contrast
pCASL: pseudo-continuous arterial spin labeling
PET: positron emission tomography
RA: room air
ROI: region-of-interest
SNR: signal-to-noise ratio
SPECT: single positron emission computed tomography
SPM: Statistical Parameter Mapping
SSS: superior sagittal sinus
TE: echo time
TI: inversion time
TR: repetition time
UTD: University of Texas at Dallas
vCBV: venous cerebral blood volume
V_enc_: velocity encoding
WB: whole-brain
WM: white matter.
